# Tranglutaminase 2 contributes to the asthmatic inflammation by modulating activation of alveolar macrophages

**DOI:** 10.1002/iid3.442

**Published:** 2021-05-04

**Authors:** Hyun Seung Lee, Da‐Eun Park, Boram Bae, Keunhee Oh, Jae Woo Jung, Dong‐Sup Lee, In‐Gyu Kim, Sang‐Heon Cho, Hye‐Ryun Kang

**Affiliations:** ^1^ Institute of Allergy and Clinical Immunology Seoul National University Medical Research Center Seoul Korea; ^2^ Department of Biomedical Sciences, Laboratory of Immunology and Cancer Biology Seoul National University College of Medicine Seoul Korea; ^3^ Department of Internal Medicine Chung‐Ang University College of Medicine Seoul Korea; ^4^ Department of Biochemistry and Molecular Biology Seoul National University College of Medicine Seoul Korea; ^5^ Department of Internal Medicine Seoul National University College of Medicine Seoul Korea

**Keywords:** asthma, macrophage, macrophage activation, tranglutaminase 2

## Abstract

**Background:**

Transglutaminase 2 (TG2), a multifunctional calcium‐dependent acyltransferase, is upregulated in asthmatic airways and reported to play a role in the pathogenesis of allergic asthma. However, the underlying mechanism is not fully understood.

**Objective:**

To investigate the role of TG2 in alternative activation of alveolar macrophages by using murine asthma model.

**Methods:**

TG2 expression was assessed in induced sputum of 21 asthma patients and 19 healthy controls, and lung tissue of ovalbumin (OVA)‐induced murine asthma model. To evaluate the role of TG2 in asthma, we developed an OVA asthma model in both TG2 null and wild‐type mice. The expression of M2 macrophage markers was measured by fluorescence‐activated cell sorting (FACS) after OVA sensitization and challenge. To evaluate the effect of TG2 inhibition in vitro, interleukin 4 (IL‐4) or IL‐13‐stimulated expression of M2 macrophage markers was measured in CRL‐2456 cells in the presence and absence of a TG2 inhibitor.

**Results:**

The expression of both TG2 and M2 markers was increased in the sputum of asthmatics compared with that of healthy controls. The expression of TG2 was increased in macrophages of OVA mice. Airway hyperresponsiveness, and the number of inflammatory cells, including eosinophils, was significantly reduced in TG2 null mice compared with wild‐type mice. Enhanced expression of M2 markers in OVA mice was normalized by TG2 knockout. IL‐4 or IL‐13‐stimulated expression of M2 markers in alveolar macrophages was also attenuated by TG2 inhibitor treatment in vitro.

**Conclusion:**

Our results suggest that TG2‐mediated modulation of alveolar macrophage polarization plays important roles in the pathogenesis of asthma.

## Graphical abstract

Transglutaminase 2 (TG2) is crucial in the development of asthma by alternatively activating alveolar macrophage as well as augmentation of Th2 response. TG2 inhibitor directly suppresses Th2 cytokine‐driven alternative activation of alveolar macrophage.

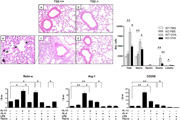

## 1 INTRODUCTION

Allergic asthma is a chronic inflammatory disease of the airways characterized by airway hyperresponsiveness (AHR), pulmonary eosinophilia, and increased release of Th2 cytokines.^1^, ^2^, ^3^

Asthma pathophysiology involves alternative activation of macrophages by Th2 cytokines, such as interleukin 4 (IL‐4) or IL‐13. Alternatively activated macrophages (M2 macrophages) express several proallergic factors, such as CD206, Ym‐1,^4^ Relm‐α, and chemokines such as macrophage inflammatory protein (MIP)‐1α, MIP‐1β, and MIP‐2,^5^, ^6^ which contribute to airway inflammation and remodeling in asthma.^7^, ^8^

Transglutaminase 2 (TG2) is a posttranslational protein‐modifying enzyme having transamidation activity and mediate crosslinking or polyamination. Several studies have shown that TG2 is abundantly expressed in endothelial cells, macrophages, and smooth muscle cells,^9^ and that it is involved in several inflammatory diseases, such as celiac disease, arthritis, and lupus.^10^ Once activated by tissue injury, TG2 induces inflammatory and fibrogenic responses.^11^, ^12^, ^13^, ^14^, ^15^ Although the expression of TG2 is upregulated by Th2 cytokines in M2 macrophages,^16^ little is known about the role of TG2 in macrophage activation in asthmatic airways.

In this study, we observed increased expression of TG2 and M2 macrophage markers in human and murine asthmatic airways. Moreover, TG2 deficiency attenuated the expression of profibrotic genes and leukotriene synthesis. We propose that the role of TG2 in alternative activation of macrophages in asthma as its proasthmatic effect.

## 2 MATERIALS AND METHODS

### 2.1 Animals

TG2 null mice (obtained from De Laurenzi and Melino^17^), and wild‐type (WT) C57BL/6J mice (purchased from the Japan SLC Inc.) were maintained at the animal facility of Seoul National University College of Medicine. Ten‐week‐old mice were used in the experiment, which was approved by the Institutional Animal Care and Use Committee (IACUC) of the Institute of Laboratory Animal Resources at Seoul National University (SNU‐10‐0216‐4).

### 2.2 Sensitization and airway challenge

Mice were sensitized by intraperitoneal injection of 2 mg alum (Pierce) and 75 μg of ovalbumin (OVA; Grade V, Sigma Aldrich) in 200 μl of phosphate‐buffered saline (PBS) on Days 0 and 7. Intranasal injection of 50 μg OVA in 20 μl of PBS was administered on Days 21, 22, and 23.

### 2.3 Measurement of methacholine AHR

AHR was measured using a barometric plethysmographic chamber (OCP 3000, Allmedicus), which provides a noninvasive measure of airway responsiveness in mice,^18^ and mice were exposed to increasing doses of methacholine using an ultrasonic nebulizer (Pari). Penh (enhanced pause), a calculated number based on inspiratory and expiratory times and pressures were monitored for 3 min, 24 h after the last OVA challenge.

### 2.4 Analysis of bronchoalveolar lavage (BAL) and serum

Mice were sacrificed 24 h after the assessment of AHR, and BAL fluid cells and lung tissue were obtained, as previously described.^8^ BAL cell slides were stained with Diff‐Quik (Sysmex Co.) and at least 300 cells per slide were evaluated to obtain a differential leukocyte count. The level of IL‐5 (Quantikine, R&D Systems Inc.), sPLA_2_ activity, and cysteinyl leukotriene (CysLT) level (Cayman Chemical) in BAL fluid was measured using a commercially available ELISA Kit, according to the manufacturer's instructions. Serum OVA‐specific IgG1, IgG2a, and IgE antibodies (Southern Biotech) were measured by using enzyme‐linked immunosorbent assay (ELISA).

### 2.5 Histopathology and immunohistochemistry

To evaluate and compare the severity and character of pathological changes in the lung parenchyma, left lungs of TG2 null mice and WT mice were fixed in 10% neutral buffered formalin and embedded in paraffin, from which 3‐mm sections were cut and stained with hematoxylin & eosin (H&E) and periodic‐acid schiff (PAS) stain. Anti‐TG2 antibody (1:2000, Abcam) was used as an immunohistochemical stain.

### 2.6 Quantitative real‐time polymerase chain reaction (qRT‐PCR)

Total RNA was extracted from the whole lung and 2 µg of each sample was reverse‐transcribed into complementary DNA (cDNA) with a cDNA synthesis kit (Promega). The mRNA expression of cytokines was determined by qRT‐PCR (Applied Biosystems), using SYBR Green Master Mix (Applied Biosystems). The expression of each gene was normalized against β‐actin and expressed relative to the control sample using the 2^‐ΔΔCt^ method, whereby ΔΔ*C*_t_ = (*C*_t_ mRNA−*C*_t_ β‐actin). All used primer sequences were validated by Primer bank.

### 2.7 Cell culture

CRL‐2456, alveolar macrophage cell line, was purchased from the American Type Culture Collection (ATCC). The cells were plated in a 24‐well plate (5 × 10^5^ per well) 1 day before exposure and then stimulated by lipopolysaccharide (LPS; 1 mg/ml, Sigma Aldrich), or recombinant IL‐4 (20 ng/ml, eBioscience) and recombinant IL‐13 (20 ng/ml, eBioscience), and cultured with or without the presence of TG2 inhibitor (100 μg/ml, B003, Zedira). Western blot was performed after 20 min incubation, using the following primary antibodies: Phospho‐STAT6 (Try641), STAT6 (1:500, Santacruz), and β‐actin (1:5000, Abcam) mRNA expression was determined by qRT‐PCR after 18 h of incubation.

### 2.8 Flow cytometry

Single‐cell suspensions of lung tissue were preincubated with FcγR‐specific blocking monoclonal antibody (2.4G2) and washed before staining. To quantify Th2 cells, single cells prepared from lung tissue were stimulated with phorbol 12‐myristate 13‐acetate (100 ng/ml), ionomycin (1 μg/ml), and Golgi stop, then pooled cells from mouse per group were stained with phycoerythrin/cy5 (PE/cy5)‐conjugated anti‐CD3 antibody and fluorescein isothiocyanate (FITC)‐conjugated anti‐CD4, PE‐conjugated anti‐IL‐13, or PE‐conjugated anti‐IL‐4 antibodies. For analysis of macrophages population, cells were stained with the following antibodies: Percp‐cy5‐conjugated anti‐CD45 (eBioscience), FITC‐conjugated anti‐F4/80 (eBioscience), allophycocyanin (APC)‐cy7‐conjugated anti‐CD11c (eBioscience), goat anti‐CD206 (R&D Systems Inc.), rabbit anti‐Ym‐1 (Stem Cell Technologies), APC‐conjugated anti‐goat IgG, or PE‐conjugated antirabbit IgG (eBioscience). Cells were analyzed on an LSRII flow cytometer (BD Biosciences) using FlowJo version 10 software (TreeStar, Inc.).

### 2.9 Inclusion criteria of the study subjects and analysis of the induced sputum

A total of 41 nonsmoking subjects (21 asthmatics and 19 asymptomatic healthy controls) without a history of corticosteroid use were included in this study. The diagnostic criteria for bronchial asthma included AHR (provocative concentration of methacholine causing a 20% fall in forced expiratory volume in one second (FEV_1_) (PC_20_) ≤ 16 mg/ml) or reversibility of bronchoconstriction (increase in FEV_1_ of ≥12% and ≥200 ml from baseline after salbutamol inhalation). Demographic characteristics, pulmonary function, and serum IgE of the study subjects were recorded. The study protocol was approved by the institutional review board of the Seoul National University Hospital (IRB No. 1610‐062‐799).

Induced sputum was processed as previously described.^19^ The cell pellet was mixed with TRIzol (Gibco) to extract the RNA before RT‐PCR was performed using a RT‐PCR kit (Promega). The mRNA of TG2, CD206, macrophage galactose‐type C‐type lectin 1 (MGL), and Arg1 was amplified on an ABI 7500 real‐time PCR system, using SYBR Green master mix (Applied Biosystems).

### 2.10 Statistical analyses

Results are expressed as mean ± standard deviation (*SD*). Kruskal–Wallis test was used to compare the groups. Where significant differences were found, Mann–Whitney test was additionally employed. Statistical analyses were performed using GraphPad Prism 4.01 (GraphPad Software). *p* ≤ .05 were considered statistically significant.

## 3 RESULTS

### 3.1 Expression of TG2 and M2 macrophage markers in induced sputum of asthmatic patients

**Figure 1:**
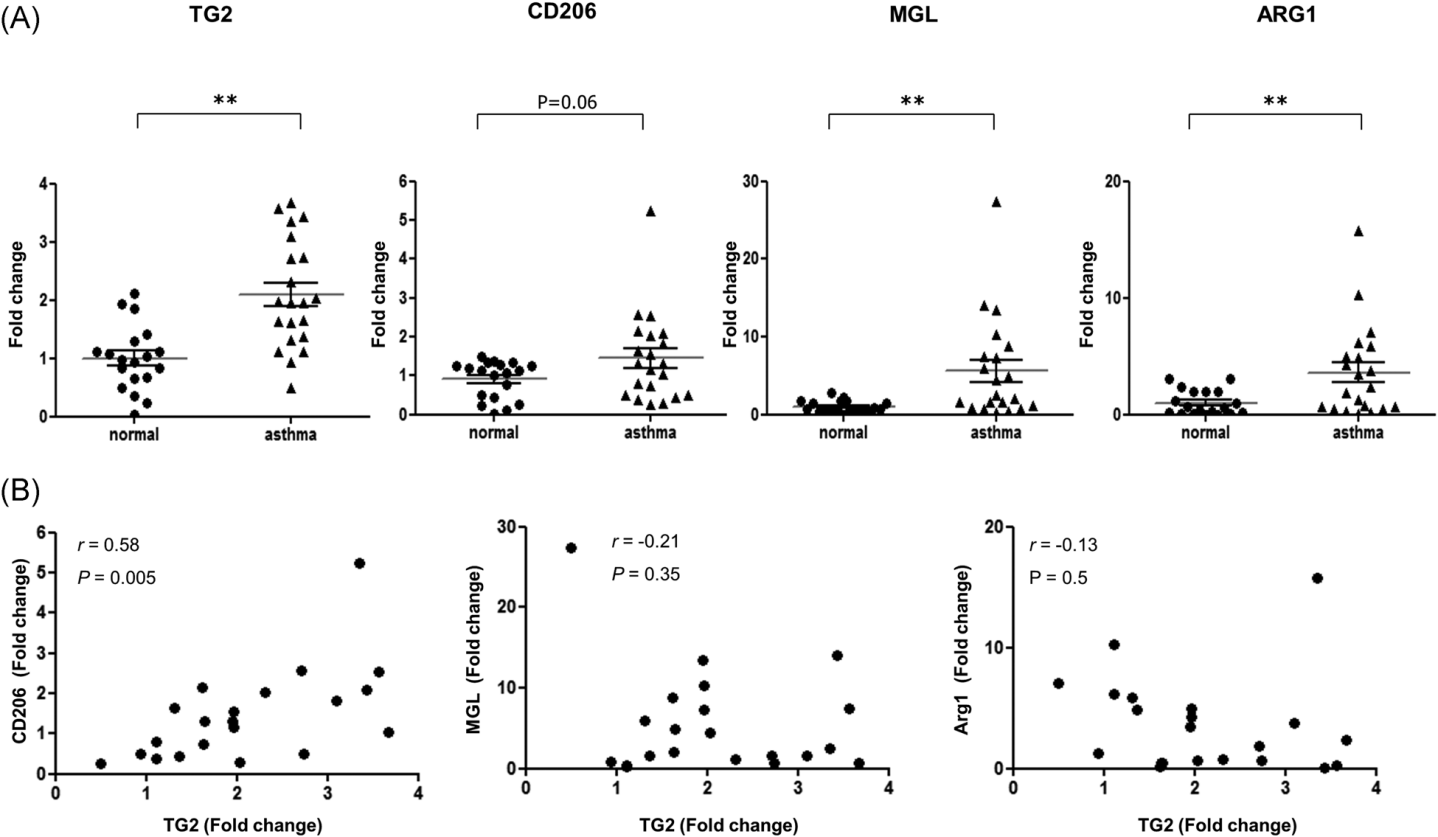
mRNA expression of TG2 and M2 macrophage markers in human induced sputum samples. The mRNA levels of TG2, CD206, MGL, and ARG1 in induced sputum of asthmatic patients compared with those of healthy controls (A, 21 asthmatics and 19 asymptomatic healthy controls). Fold change values presented in the *Y*‐axis of each graph. Correlation between sputum CD206 and TG2 expression, between sputum MGL and TG2 expression, and between sputum Arg1 and TG2 expression (B). Correlation coefficients (*r*) were measured using Pearson's method. MGL, macrophage galactose‐type C‐type lectin 1; TG2, Transglutaminase 2. *p < .05. **p < .01

First of all, to determine the difference in expression of TG2 in normal and asthmatic patients, its expression in sputum was examined. As a result, the mRNA expression of TG2 was twofold higher in the asthma group than in the control group (Figure 1A). CD206 expression, known as one of the representative markers of M2 macrophage, was borderline increased in the asthma group compared with the control group (*p* = .06), and showed a significant correlation with TG2 expression (*r* = .58, *p* = .005) (Figure 1B). The mRNA levels of MGL and ARG1 were also significantly higher in the asthma group than in the control group (Figure 1A), but they were not significantly correlated with sputum TG2 expression in asthma patients (Figure 1B).

### 3.2 Airway inflammation and AHR in TG2 null mice

**Figure 2:**
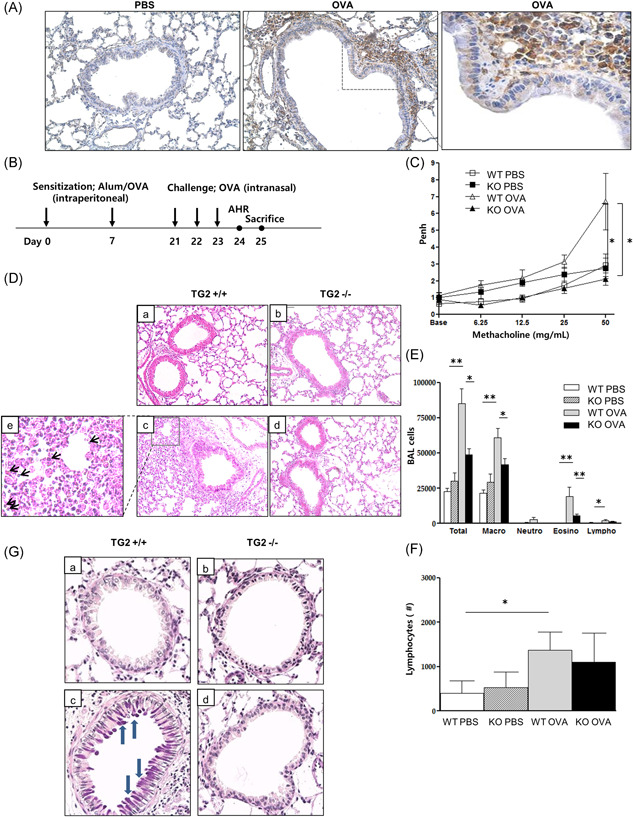
TG2 expression and the effect of TG2 on lung inflammation and AHR following allergen challenge. TG2 expression in the lung of a murine asthma model (×400) (A). Experimental protocol of the study (B, *n* = 5–6 mice per group). Methacholine hyperresponsiveness was measured 24 h after the final intranasal OVA challenge. AHR was expressed as the enhanced pause (Penh) (C). H&E‐stained lung histology after allergen challenge from the mice of the different groups (a: WT PBS, b: KO PBS, c: WT OVA, d: KO OVA, e: black arrows indicate eosinophils, ×200) (D). The number of inflammatory cells, including eosinophils (E) and lymphocytes (F) in BAL fluid. PAS‐stained lung histology after allergen challenge from mice of the different groups (a: WT PBS, b: KO PBS, c: WT OVA, d: KO OVA, blue arrows indicate mucin secretion, ×400) (G). AHR, airway hyperresponsiveness; BAL, bronchoalveolar lavage; H&E, hematoxylin and eosin; KO, knockout; OVA, ovalbumin; PAS, periodic‐acid schiff; PBS, phosphate‐buffered saline; TG2, transglutaminase 2; WT, wild type. *p < .05, **p < .01.

To evaluate the expression of TG2 in OVA‐induced asthma mice, immunohistochemistry staining was performed on the lung tissue of the mice. TG2 staining showed that TG2 expression was prominent in peribronchial macrophages rather than airway epithelium of the OVA mouse model (Figure 2A). Since the increased expression of TG2 in OVA‐induced asthma mice was confirmed, an experiment was conducted with TG2 null mice to examine the role of TG2 (Figure 2B). In WT OVA mice, AHR and airway inflammation including macrophages, eosinophils, lymphocytes, as typical phenotypes of asthma, were increased compared with WT PBS (Figure 2C−F). On the other hand, in the experiment using TG2 null mice, AHR was significantly reduced in the absence of TG2 (Figure 2C) and the numbers of inflammatory cells, including macrophages and eosinophils, were reduced in TG2 null mice compared with WT OVA mice (Figure 2C−E). Meanwhile, it was also performed PAS staining to access the mechanism of airway hypersensitivity. As a result, mucin secretion was increased in WT OVA mice compared with WT PBS. However, mucin secretion was decreased in TG2 null mice compared with WT OVA mice (Figure 2G).

### 3.3 Th2 responses, profibrotic gene expression, and leukotriene levels in TG2 null mice

**Figure 3:**
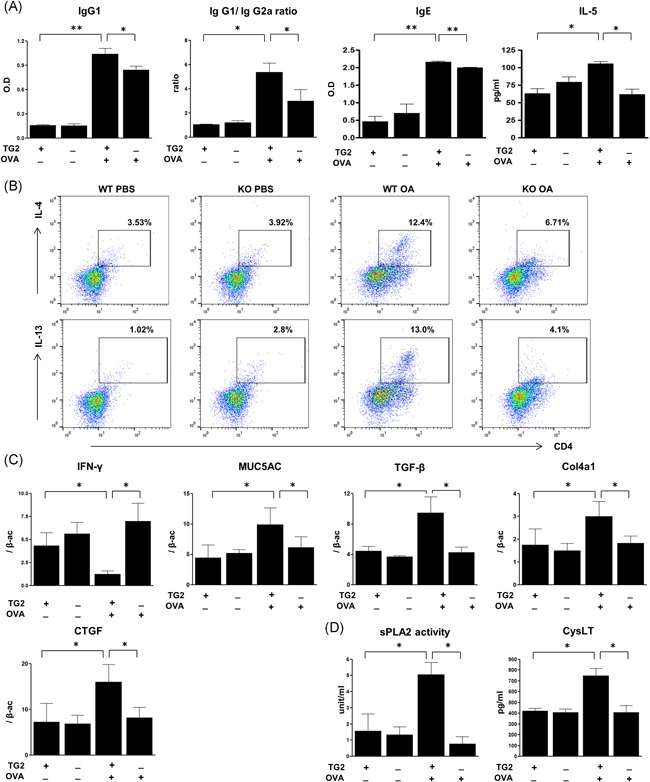
Effects of TG2 on serum allergen‐specific antibodies, cytokines, and mRNA expression in mouse lung tissue. Serum OVA‐specific IgG1, IgG1/IgG2a ratio, IgE, and IL‐5 in BAL fluid after OVA challenge (A). IL‐4 (upper panel) or IL‐13 (lower panel)‐produced CD4^+ ^in CD3^+ ^cells were evaluated from pooled lung per group (*n* = 5–6 mice per group) using flow‐cytometry (B). % indicates the population in CD3^+^ cells. Real‐time PCR was performed to monitor the changes in mRNA levels in lung tissue. The expression of IFN‐γ, MUC5AC, TGF‐β, and Col4a1 mRNA evaluated after the OVA challenge (C). The levels of mRNA are expressed as a ratio with β‐actin. PLA_2_ activity and CysLT level were measured in BAL fluid (D). BAL, bronchoalveolar lavage; IL‐4, interleukin‐4; OVA, ovalbumin; CysLT, cysteinyl leukotriene; TG2, transglutaminase 2. *p < .05, **p < .01.

The levels of serum OVA‐specific IgG1, IgG1/G2a ratio, and IgE were significantly reduced in TG2 null mice compared with WT OVA mice. IL‐5 level in BAL fluid was decreased in TG2 null mice compared with WT OVA mice (Figure 3A). As a result of examining IL‐4 and IL‐13 in CD4^+^cells from the pooled mouse lung tissue per group, these populations were increased in WT OVA mice compared with WT PBS mice. However, the populations of IL‐4 and IL‐13 were reduced in TG2 null mice compared with WT OVA mice (Figure 3B). Meanwhile, the mRNA expression of interferon‐γ (IFN‐γ) in lung tissue was increased in TG2 null mice compared with WT OVA mice (Figure 3C).

The lungs of WT OVA‐challenged mice had increased mRNA levels of MUC‐5AC, TGF‐β, Col4a1, and CTGF compared with those of control mice. TG2 knockout was able to normalize these mRNA expression levels (Figure 3C). sPLA_2_ activity and CysLT level were significantly lower in BAL fluid of TG2 null mice compared with that of WT OVA mice (Figure 3D).

### 3.4 Expression of M2 macrophage markers in lung homogenates

**Figure 4:**
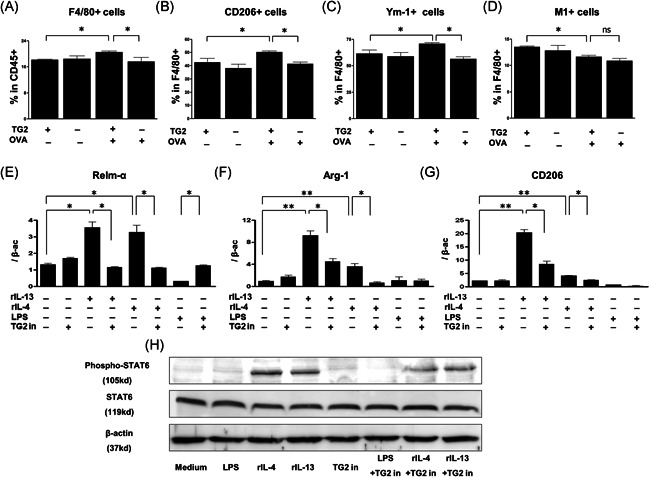
Effects of TG2 on the expression of M2 macrophage markers in mouse lung tissue and alveolar macrophages. (A–D) The expression of M2 macrophage markers was measured by FACS in TG2 null mice and WT mice after OVA sensitization and challenge (*n* = 5‐6 mice per group). The proportion of F4/80^+^ macrophages in CD45^+^ cells (A), F4/80+ macrophage expressing CD206 (B), Ym‐1 (C), and CD11c^+^CD206^−^ (M1) (D) are depicted. The mRNA levels of Relm‐α (E), Arg‐1 (F), and CD206 (G) in Th2 cytokine (20 ng/ml)‐stimulated mouse alveolar macrophages were compared according to the TG2 inhibitor treatment (100 μg/ml). Experiments were performed at least three times. The levels of mRNA are expressed as a ratio with β‐actin. Western blot analysis of phospho‐STAT6, total STAT6, and β‐actin expression in Th2 cytokine‐stimulated mouse alveolar macrophages were compared with or without a TG2 inhibitor (H). % Area of phospho‐STAT6 was measured by ImageJ. FACS,  fluorescence‐activated cell sorting; OVA, ovalbumin; WT, wild type; STAT, signal transducer and activator of transcription; Tg2, transglutaminase 2. *p < .05, **p < .01

F4/80 expression in CD45^+^ lung cells increased in WT OVA‐challenged mice, and significantly reduced in TG2 null mice (Figure 4A). Similarly, higher expression levels of CD206 and Ym‐1 in F4/80^+^ macrophages were observed in WT OVA‐challenged mice, while expression of these markers was significantly decreased in TG2 null mice (Figure 4B,C). In contrast, M1 macrophages (identified by gating on F4/80^+^CD11c^+^CD206^−^ cells^20^, ^21^) were reduced in OVA‐challenged mice compared with control mice, but their number was not affected by TG2 deficiency (Figure 4D).

We also examined the in vitro effects of TG2 inhibition on Th2 cytokine‐stimulated M2 macrophage markers, in CRL‐2456 macrophages. The mRNA expression of Relm‐α, Arg‐1, and CD206 significantly decreased in IL‐4‐ or IL‐13‐stimulated CRL‐2456 macrophages exposed to the TG2 inhibitor (Figure 4E‐G), but not in those stimulated with LPS.

Western blot analysis revealed that IL‐4 and IL‐13‐induced increase in phosphorylated STAT6 was attenuated with TG2 inhibitor treatment (Figure 4H).

## 4 DISCUSSION

TG2 plays a crucial part in a number of inflammatory diseases. Previous studies reported upregulation of TG2 in human asthmatic airways^22^, ^23^; in patients with exercise‐induced bronchoconstriction, TG2 expression was correlated with disease worsening.^22^, ^23^ Toluene diisocyanate, a well‐known inducer of occupational asthma, seems to activate TG2 in airway epithelial cells, resulting in airway inflammation.^23^ In a murine asthma model, TG2 is expressed in airway epithelium where it triggers inflammation and airway remodeling by acting on the PLA2‐cysteinyl‐LTs axis.^24^ TG2 stimulates nuclear factor‐κB activation in lung epithelial cells of OVA‐challenged mice, and TG2 inhibition alleviates airway inflammation of allergic asthma in a murine asthma model.^25^ It was previously shown that asthmatic phenotypes, such as AHR and eosinophilic inflammation, were diminished in TG2‐deficient OVA‐challenged mice and that pulmonary epithelial cells damaged by allergens induced TG2‐mediated IL‐33 expression, which activated both the innate and adaptive immune responses.^26^ In this study, we conducted alum/OVA‐induced asthma mouse model. The alum/OVA mouse model is well known as a typical method of inducing a systemic asthma model.^27^ OVA is well‐known allergen used to induce murine asthma model. Aluminum hydroxide (alum adjuvant) is administered with OVA during the sensitization period to induce a systemic model, and sensitization is administered intraperitoneal twice. After that, if the challenge process is undergone with intranasal OVA administration, humoral immune responses (immunoglobulin production via Th2 stimulation) and immune cells are activated.^28^

In this study, deletion of TG2 in OVA‐challenged mice diminished AHR, airway inflammation, Th2 responses, profibrotic gene expression, and leukotriene level.

The above‐mentioned studies demonstrated that TG2 secreted by pulmonary epithelial cells plays an important part in airway remodeling and the allergic Th2 response. However, the role of TG2 secreted by other types of cells is not well understood. In this study, the expression of TG2 was rather prominent in lung macrophages than in epithelial cells of a murine asthma model. Besides, we observed increased TG2 expression in induced sputum samples of asthma patients compared with those of healthy controls. Since the inflammatory cells of induced sputum are mostly macrophages, it is highly likely that TG2 in these samples originated from alveolar macrophages. As for the relationship between TG2 and macrophages, TG2 was previously reported to be directly released from Th2 cytokine‐stimulated macrophages, as known as M2 macrophages, in both mouse and human cells.^16^ Our data also showed that CD206 expression, a representative marker of M2 macrophages in human^29^ has significant correlation with TG2 expression. Although M2 macrophage subsets are usually classified as M2a, M2b, and M2c in mice, M2 subtypes are not clearly characterized in human. The good correlation of TG2 with CD206 but not with MGL or ARG1 suggests that TG2 and CD206 might belong to the same M2 subtype in association with asthma pathogenesis.

Th2 cytokines, such as IL‐4 or IL‐13, contribute to the development of inflammation in the lungs of OVA‐induced murine asthma model by promoting differentiation of macrophages into alternatively activated M2 macrophages.^30^ A significant positive correlation was observed between the number of type 2 innate lymphoid cells (ILC2s) and the number of M2 macrophages in induced sputum of asthmatic patients, and the expression of M2 macrophage‐related genes was enhanced by ILC2.^31^ A nonspecific chemical stimulus, such as chloride exposure, can aggravate Th2 inflammation by enhancing both ILC2 and M2 in a murine asthma model. Transfer of alternatively activated M2 subsets into control mice resulted in AHR, enhanced Th2 cytokine secretion, and eosinophilic inflammation.^32^ In addition, Ym‐1 released from M2 macrophages directly accelerated Th2 responses^33^ in parasite‐activated, Ym1‐expressing macrophages. We previously reported that the anti‐asthmatic effect of thalidomide is mediated by inhibition of M2 polarization.^34^ Owing to their M2 suppressing effect, TG2 inhibitors could be potential anti‐asthmatic agents.

CD206, Relm‐α, Ym‐1, and Arg‐1 are known M2 macrophage markers.^35^ CD206 (macrophage mannose receptor 1), a well‐known marker of alternative macrophage activation, is implicated in airway remodeling and its levels are increased in alveolar macrophages of patients with idiopathic pulmonary fibrosis.^36^ Arg‐1 expression is upregulated in the lungs of allergen‐induced mice, and disruption of its expression is associated with a reduction of airway responsiveness.^37^ Relm‐α also contributes to the pathogenesis of allergic airway disease via airway remodeling.^38^, ^39^ In this study, the expression of Ym‐1 and CD 206, the major indicators of alternatively activated macrophages, was clearly impaired in TG2 null OVA‐challenged mice. However, the population of M1 macrophages did not show any remarkable changes in response to TG2 modulation. To directly assess the effect of TG2 on macrophage activation, alveolar macrophages were exposed to a TG2 inhibitor, which suppressed IL‐4 or IL‐13‐induced Relm‐α, Arg‐1, and CD206 expression. Thus, we propose that TG2 is implicated in autocrine regulation of M2 macrophage polarization.

On the other hand, CysLTs produced from eosinophils, mast cells, and macrophages in addition to epithelial cells are involved in the airway inflammation and AHR.^40^ Along with TG2 regulation of CysLTs in epithelial cells of asthmatic airways, TG2 can also regulate the production of CysLTs in macrophage. In this study, both sPLA_2_ activity and CysLT level were significantly lower in BAL fluid of TG2 null mice compared with that of WT OVA mice, which suggested that CysLT secretion may be affected by TG2 deletion in macrophages. In accordance with, the production of CysLTs in IL‐4‐treated macrophages was effectively reduced by TG2 inhibition in the previous study.^41^

STAT6 activation is required for the development of allergic asthma.^42^ STAT6 is a Th2‐associated transcription factor that is activated through phosphorylation and translocates into the nucleus during M2 activation.^43^, ^44^ The differentiation of M2 macrophages is induced by IL‐4‐and IL‐13‐dependent activation of STAT6, resulting in the expression of M2 markers such as CD206 and Arg1 in macrophages in vitro.^45^, ^46^ In this study, TG2 secreted by M2 macrophages regulated M2 polarization by activation of STAT6, while TG2 inhibition reduced STAT6 phosphorylation. The ability of TG2 inhibitors to block STAT6 activation and impair M2 differentiation suggests that TG2 inhibitors might exhibit anti‐asthmatic activity.

Our results show that TG2 inhibition suppresses not only Th2 responses but also macrophage activation, highlighting a potential mechanism underlying the anti‐asthmatic effect of TG2 inhibitors. M2 macrophages also play an important role in the proliferation of CD4^+^ lymphocytes in asthmatic patients.^47^ Upregulated MGL1 in alveolar macrophages of asthmatic patients^48^ recognizes terminal galatose or *N*‐acetylgalactosamine residues present on naturally arising allergens.^49^, ^50^ Moreover, the mRNA expression of Arg‐1 is upregulated in human submucosal inflammatory cells.^51^ M2 macrophage‐derived arginase‐1 catalyzes the breakdown of arginine into prolines and polyamines essential for collagen synthesis while M2 macrophage‐derived Relm‐α induces collagen production and differentiation of myofibroblasts.^52^ This study showed that the expression of profibrotic genes was reduced in TG2 null OVA‐challenged mice. Therefore, we suggest that M2 macrophages can modulate profibrotic gene expression. One of the mechanisms of reduced AHR in TG2 null mice in this study could be a reduction in mucin secretion since excessive M2 macrophages may increase mucus secretion, resulting in AHR.^53^

This study revealed that TG2 may affect the pathogenesis of asthma by regulating M2 macrophages by using conventional KO mice. However, to clearly differentiate and give an insight on the specific role of epithelial TG2 versus macrophage TG2, a further study using conditional TG2 mice is required.

In conclusion, this study found that TG2 contributes to the development of allergic airway inflammation and remodeling by increasing OVA‐specific sensitization and expression of profibrotic and proallergic mediators related to allergic Th2 inflammation. TG2 expression in macrophages is directly related to alternative activation of macrophages via STAT6 signaling.

## CONFLICT OF INTERESTS

The authors declare that there are no conflict of interests. 

## AUTHOR CONTRIBUTIONS

Hyun Seung Lee and Hye‐Ryun Kang conceived and designed the study. Keunhee Oh, Jae Woo Jung, Dong‐Sup Lee, In‐Gyu Kim, and Sang‐Heon Cho supervised the research. Hyun Seung Lee, Da‐Eun Park, and Boram Bae performed the experiments. Hyun Seung Lee, Da‐Eun Park, Boram Bae, and Hye‐Ryun Kang analyzed the data. Hyun Seung Lee and Hye‐Ryun Kang wrote the manuscript.

## Data Availability

The data that support the findings of this study are available from the corresponding author upon reasonable request.
